# Metasurface-enabled three-in-one nanoprints by multifunctional manipulations of light

**DOI:** 10.1016/j.isci.2021.103510

**Published:** 2021-11-26

**Authors:** Zile Li, Liangui Deng, Juan Deng, Zhixue He, Jin Tao, Guoxing Zheng, Shaohua Yu

**Affiliations:** 1 Electronic Information School, Wuhan University, Wuhan 430072, China; 2 Peng Cheng Laboratory, Shenzhen 518055, China; 3 State Key Laboratory of Optical Communication Technologies and Networks, China Information Communication Technologies Group Corporation (CICT), Wuhan 430074, China; 4 Suzhou Institute of Wuhan University, Suzhou 215123, China

**Keywords:** Optics, Applied physics, Engineering

## Abstract

In metasurface-based ultra-compact image display, color-nanoprints, gray-imaging elements, and binary-pattern-imaging elements are three different types of nanoprints, implemented with different mechanisms of light manipulation. Here, we show the three functional elements can be integrated together to form a “three-in-one” nanoprint with negligible crosstalk, merely with a single-cell nanostructured design approach. Specifically, by decoupling spectrum and polarization-assisted intensity manipulations of incident light, the proposed metasurface appears as a dual-color nanoprint under a broadband unpolarized light source illumination, while simultaneously displaying an independent continuous gray image and another binary-pattern in an orthogonal-polarization optical setup with different polarization controls. Our approach can increase the system integration and security of metasurfaces, which can be of interest to many advanced applications such as data storage, optical information encoding, high-end optical anti-counterfeiting, and optical information hiding.

## Highlights

• Merging three types of nanoprints into one metasurface with negligible crosstalk
• A single-cell nanostructure design approach is proposed

## Graphical abstract

**Figure:**
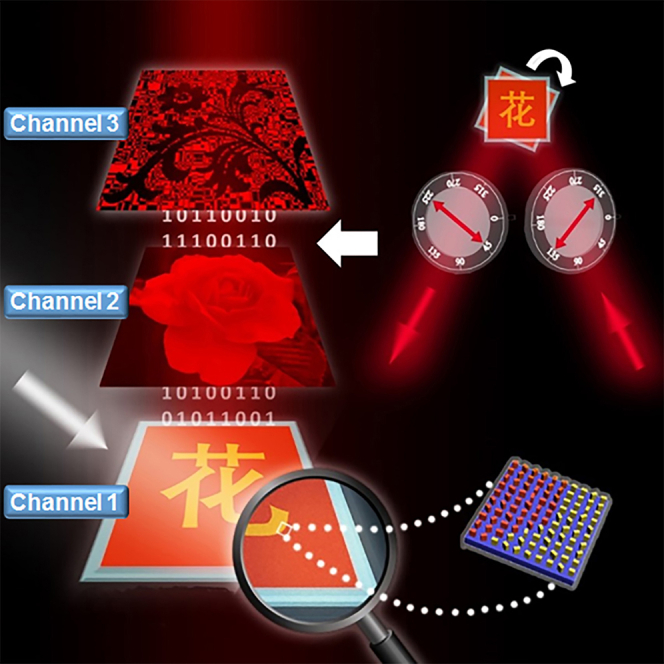

## Introduction

As a kind of artificially designed material, nanostructured metasurfaces have enabled the advanced controls of amplitude, phase, and polarization of incident light at the nanoscale. By judiciously designing the geometry and orientation of each nanostructure, metasurfaces have been employed to act as optical functional elements such as metalenses (Wang et al., 2018; Wang et al., 2021; Li et al., 2021a; Chen et al., 2018a; Zheng et al., 2017; Fu et al., 2019), meta-holograms (Li et al., 2017; Jiang et al., 2019; Li et al., 2020a; Li et al., 2020b; Fu et al., 2020; Hu et al., 2019; Deng et al., 2020a; Zhang et al., 2020; Kim et al., 2021a; Ren et al., 2020), meta-gratings (Yang et al., 2021; Li et al., 2018; Fang et al., 2020; Li et al., 2015a), vortex beam generators (Hu et al., 2021; Chen et al., 2018b; Dai et al., 2020a; Guo et al., 2016), and quantum information carriers (Solntsev et al., 2021; Zhu et al., 2020; Li et al., 2020c). Among them, metasurface-based nanoprints have attracted extensive interest due to their subwavelength resolution, durable properties, nonfading colors, and zero-pollution (Chen et al., 2019a; Yang et al., 2020; Zheng et al., 2021; Kim et al., 2021b; Chen et al., 2019b; Jung et al., 2020). Benefiting from the spectral tunability of metasurfaces, various approaches have been proposed to encode nanoprinting images with different colors onto a single metasurface. For example, by adjusting the resonant spectra in transmission or reflection mode, color meta-nanoprints based on aluminum (Xu et al., 2010; Ellenbogen et al., 2012; Tan et al., 2014; Jang et al., 2019; Olson et al., 2015; Tseng et al., 2017), silver (Li et al., 2015b; Cheng et al., 2015; Liu et al., 2019), silicon (Proust et al., 2016; Dong et al., 2017; Li et al., 2021b; Liang et al., 2021; Yoon et al., 2018), and titanium oxide (Sun et al., 2017; Li et al., 2020b; Koirala et al., 2018) nanostructures have been successively proposed. Further, by extending the spectral tunability to anisotropic nanostructures, polarization multiplexed color nanoprints are created by using metal-insulator-metal (MIM) nanoellipses (Goh et al., 2014), titanium oxide nanobricks (Yang et al., 2018), and cross-shaped aluminum nanostructures (Zhang et al., 2019). These color meta-images can be readily observed under the illumination of white light. In general, the multiplexing nanoprints aforementioned can achieve two information channels because light wave has 2 degrees of freedom for orthogonal-polarization control, e.g., linearly polarized (LP) light in x/y axes, left- and right-handed circularly polarized (LCP/RCP) light, etc.

Optical pattern can be encoded not only into color profiles but also into spatially varying intensity, i.e. grayscale modulation. Inspired by the Malus law, researchers have proposed the polarization-controllable image display technique, with which one can utilize nanostructures acting as half-wave plates (Yue et al., 2018; Shan et al., 2020) or polarizers (Li et al., 2021c; Deng et al., 2019; Deng et al., 2020b; Dai et al., 2020b; Guo et al., 2019) to construct gray-imaging elements with ultra-high resolution and extraordinary ability of continuous grayscale modulation. In addition, in some applications such as quick response (QR) code for information recognition and watermark for anti-counterfeiting, binary patterns are more suitable for information encoding. Recently, multiplexing grayscale or binary-pattern nanoprints have been proposed by finely setting the size or orientation of nanostructures (Dai et al., 2020a, 2020b, 2020c; Deng et al., 2020c; Fan et al., 2020; Liu et al., 2021), which further improve the density of information storage.

Merging a color-nanoprint, a gray-imaging element and a binary-pattern-imaging element into a single metasurface are an artful approach to increase the information security and system integration, which can also provide a new information multiplexing method. However, different types of nanoprints always correspond to different light control mechanisms. The difficulty of realizing multifunctional manipulations of light hinders the development of “three-in-one” nanoprints. In this paper, we show a route of integrating color and grayscale manipulations into a single metasurface and control them separately to form different information channels, which enables the concept of “three-in-one” nanoprint, simply by a single-cell design approach. Specifically, based on the spectral differences of two dielectric nanobricks with different dimensions, a dual-color nanoprinting image can be recorded right at the metasurface plane. At the same time, the two different nanobricks have equal polarization conversion efficiency (PCE) near the designing wavelength of 610 nm, which ensures that they can produce an equal intensity governed by Malus law. Based on this characteristic, a continuous grayscale image can be encoded into the dual-color nanoprint. Interestingly, inspired by the orientation degeneracy of anisotropic nanostructures, the same metasurface can simultaneously record an additional binary-pattern, merely with polarization controls.

**Figure 1:**
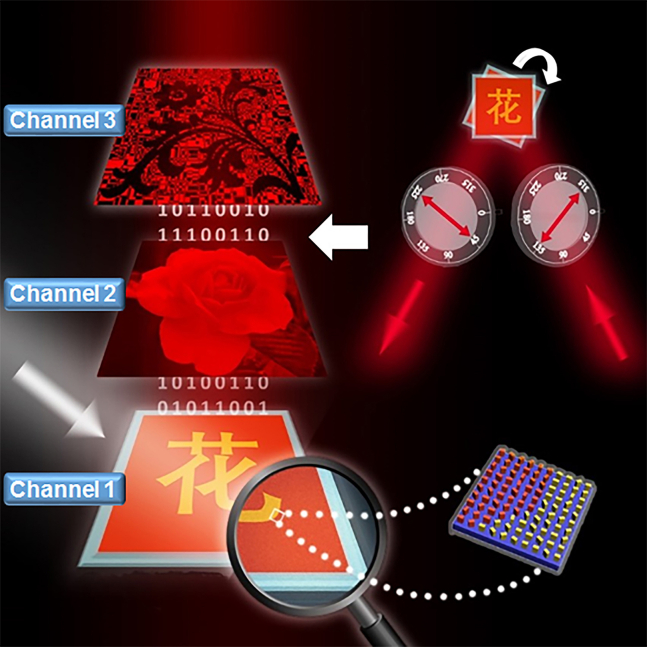
Schematic illustration of the “three-in-one” nanoprints with a single-cell-nanostructure design approach and some application prospects The metasurface is composed of two types of nanobricks with different dimensions but each unit-cell contains only one nanobrick (i.e. single-cell-nanostructure). Under the white light illumination, a dual-color image appears right at the metasurface plane (channel 1). An orthogonal-polarization optical path consisting of two bulk-optic polarizers and a narrow-band filter is taken as an optical key to decode the hidden continuous grayscale image (channel 2) and binary-pattern (channel 3).

Figure 1 shows the basis concept of the proposed metasurface. Apparently, the metasurface is a dual-color nanoprint observed under a white light illumination without polarization control. Actually, two additional information channels have been hidden into the metasurface, and the corresponding images can be decoded by utilizing specific optical keys. Specifically, when we put the metasurface into an orthogonal-polarization optical path consisting of two bulk-optic polarizers and a narrow-band filter, a continuous grayscale image can be decoded. If we rotate the metasuface around its optical axis by 22.5°, a binary-pattern appears (the two images can also be switched by rotating the two bulk-optic polarizers, as shown in Figure S1). Therefore, three different types of nanoprinting images can be recorded with a piece of metasurface. With aforementioned unique characteristics, the “three-in-one” nanoprints have potential applications in multi-folded anti-counterfeiting, optical storage, information encoding and hiding, etc.

## Results

### Design of the tri-channel metasurfaces for “three-in-one” nanoprints

To obtain the tri-channel metasurface for information multiplexing aforementioned, we need to retrieve a pair of nanostructures that have different spectral response but equal PCE at a fixed wavelength, which meets the requirement of forming a dual-color image (channel 1) under white light illumination and two gray images at a fixed wavelength. Because decoding a continuous grayscale image (channel 2) and a binary-pattern (channel 3) requires an orthogonal-polarization optical path, two bulk-optic polarizers acting as a polarizer and an analyzer, respectively, are placed before and after a nanostructure, then we can deduce the intensity after the analyzer as

(Equation 1) $$I_{1}=I_{0}{\left(\frac{A-B}{2}\right)}^{2}{\cos\;}^{2}\left(2\theta -\alpha_{2}-\alpha_{1}\right),$$

where *A* and *B* indicate the complex reflection coefficients when the light waves propagate with polarization along the long and short axes of the nanobricks, *θ* denotes the orientation angle of the nanobrick, *α*_1_ and *α*_2_ are the transmission axis directions of the polarizer and analyzer respectively, and *I*_0_ is the light intensity after the polarizer. In particular, if *α*_2_ = -*α*_1_ = 45° and *θ* = 0°, the ratio of output light intensity to the incident LP light is ${\left|\frac{A-B}{2}\right|}^{2}$, which is defined as PCE aforementioned. More details of the formula derivation are presented in theoretical analysis of STAR Methods.

Here, we employ silicon-on-insulator (SOI) materials that are widely employed in integrated circuits, to make a reflective-type all-dielectric metasurfaces. To satisfy the aforementioned conditions, we elaborately design the geometry of nanostructures by using CST Microwave Studio software. Two types of nanobricks with the equal height *H* = 220 nm and cell size *C* = 400 nm are employed in our design, named as I and II, respectively. When Nanobrick I is designed with length *L*_1_ = 150 nm and *W*_1_ = 90 nm and Nanobrick II is designed with *L*_2_ = 180 nm and *W*_2_ = 100 nm, the reflection spectra are different enough to produce two different structural colors. At the same time, the PCE of the two types of nanobricks is almost equal at a working wavelength of 610 nm (detailed description about the design and simulation of the nanobricks is provided in the numerical simulations of STAR Methods). Therefore, both of them can be employed to construct a hybrid metasurface for storage of both dual-color image and gray-images.

**Figure 2:**
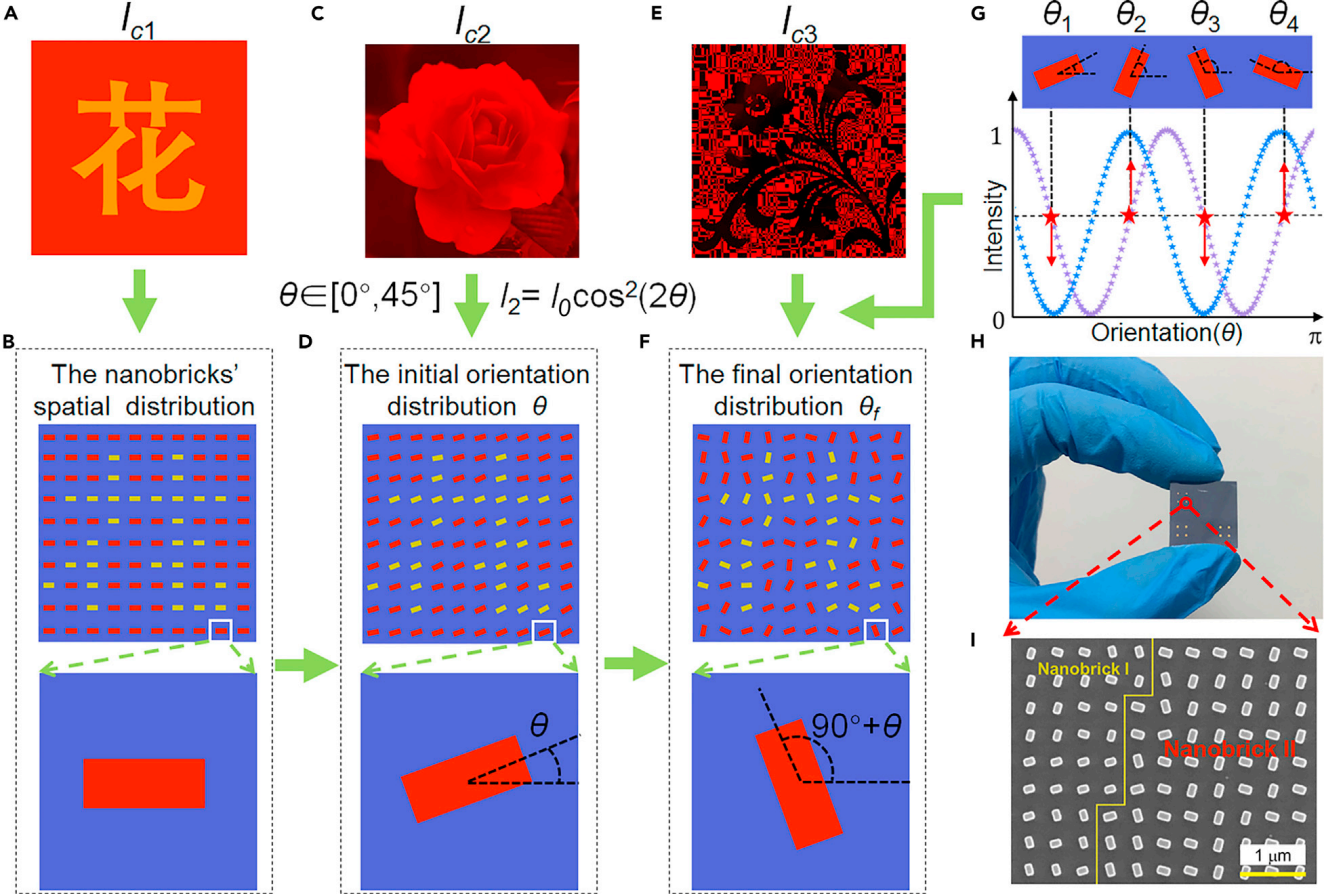
Design flowchart of the tri-channel metasurface for the integration of a dual-color image, a continuous grayscale image, and a binary-pattern (A) The target dual-color image *I*_c1_. (B) The spatial distribution of the two types of nanobricks. (C) The target gray-image *I*_c2_. (D) The initial orientation distribution *θ*. (E) The target binary image *I*_c3_. (F) The final orientation distribution *θ*_f_. (G) Illustration of the orientation degeneracy. (H and I) A photo of the fabricated metasurface sample and a scanning electron microscope (SEM) image in partial view, where two types of nanobricks (I and II) are denoted with different color words.

With the above designed two types of SOI nanobricks, we can now implement the tri-channel metasurface design, as shown in Figure 2 of the design flowchart. Because we use a single-cell design strategy, no supercell is required. In general, the tri-channel metasurface design includes two aspects: (1) spatial distribution of the two types of nanobricks with different dimensions; (2) orientation distribution of nanobricks. Firstly, we can determine the spatial distribution of the two types of nanobrick according to the target image of channel 1. The background and target parts of *I*_c1_ are designed with Nanobrick I and II, respectively, as shown in Figure 2B. Next, the target gray-image *I*_c2_ and the normalized intensity modulation of channel 2, i.e., *I*_2_ = *I*_0_cos^2^(2*θ*), are utilized to calculate the initial orientation *θ*, in which all orientations lie in the interval of [0°, 45°].

The last step is rearranging the orientations to construct channel 2 and 3 simultaneously with the help of polarization multiplexing. Specifically, if one rotates the orthogonal-polarization optical setup (two bulk-optic polarizers) clockwise from the current 0° to an angle such as 22.5°, the new light intensity can be written as *I*_3_ = *I*_0_cos^2^(2*θ*+45°). We plot both *I*_2_ and *I*_3_ versus orientation angle, as shown in Figure 2G. And we found that there exists a one-to-four mapping relationship between the light intensity and the orientation of nanobrick in the defined interval of [0°, 180°], which can be called as the orientation degeneracy of nanobricks. That is, there are four options for the orientation angles, *θ*_1_, *θ*_2_, *θ*_3_, and *θ*_4_, to generate the equal output light intensity corresponding to channel 2. However, in the intensity modulation of *I*_c3_ corresponding to channel 3, the four orientation angles possess two different intensity modulations (*θ*_1_ and *θ*_3_ correspond to a “low” intensity value [<0.5]; *θ*_2_ and *θ*_4_ correspond to a “high” intensity value [>0.5]), opening up a new design degree of freedom to create an additional “binary-pattern” without complicating the design and fabrication of nanostructures. Therefore, it is promising to search a reasonable orientation distribution that satisfies the requirement of encoding a continuous gray-image and a binary-pattern into channel 2 and channel 3, respectively. Specifically, if the intensity value of *I*_3_ is lower than 0.5, the corresponding initial orientation remains unchanged (= *θ*_1_) or is changed to *θ*_3_. If the intensity value of *I*_3_ is larger than 0.5, the corresponding initial orientation distribution *θ* is changed to be *θ*_2_ or *θ*_4_. Hence, we get the final orientation distribution *θ*_f_, as shown in Figure 2F. It is worth noting that the intensity value cannot be set to be 0 or 1 in channel 2 (in this case, the intensity value is 0.5 for each pixel in channel 3). Besides, the intensity profile in channel 3 is not a pure binary-intensity (the intensity values are modulated to be exactly 0 or 1) in traditional sense. In our work, the binary image denotes the image has two kinds of intensity value, one is higher than 0.5 and the other is lower than 0.5, so the dark (bright) part on an image is not dark (bright) enough and the contrast is not high enough compared with a traditional binary image. Therefore, there is a trade-off between encoding more images in nanoprint in a single band and generating higher contrast images.

### Experimental demonstration of the “three-in-one” nanoprints

**Figure 3:**
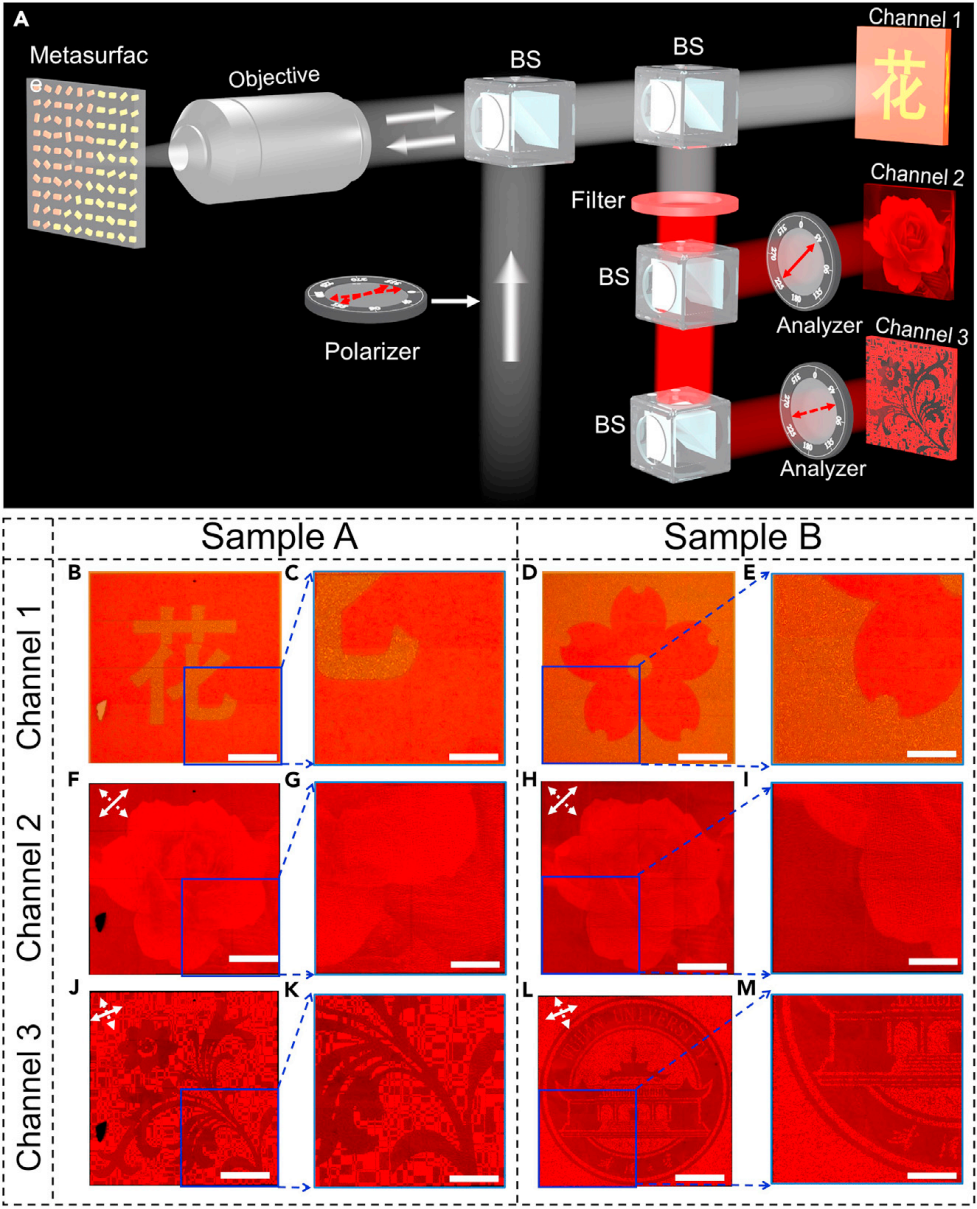
Experimental setup and results of the two metasurface samples, and each of them integrates a dual-color image, a continuous grayscale image, and a binary-pattern into a single-cell-nanostructured metasurface (A) Experimental setup to decode the three nanoprinting images. To decode the hidden gray-image and binary-pattern, a polarizer and an analyzer are utilized to construct an orthogonal-polarization optical path. BS, beam-splitter. (B–E) Dual-color nanoprinting images under a quartz halogen lamp illumination. (F–I) Experimentally captured continuous grayscale images decoded by tuning an orthogonal-polarization optical path. The two different white arrows in the upper left corner represent the transmission axis directions of the polarizer and the analyzer, respectively, i.e., *α*_1_ = −45° and *α*_2_ = 45°. (J–M) Experimentally captured binary-patterns with *α*_1_ = −22.5° and *α*_2_ = 67.5°. The scale bars are 50 μm and 25 μm in the experimental images and their zoom-in views, respectively.

To demonstrate the feasibility and flexibility of the “three-in-one” nanoprints, we fabricate two different types of samples (labeled with A and B) by using the standard electron beam lithography (see STAR Methods for details of the sample fabrication). Both samples have dimensions of 200 μm × 200 μm. Figures 2H and 2I show the photo and the SEM image of the fabricated metasurface sample. For samples A and B, we encode the same gray-image but different dual-color image and binary-pattern. In our design, the target and background parts of sample A are designed with Nanobrick I and II respectively, whereas the situation of sample B is the opposite of sample A, i.e., the target and background parts are designed with Nanobrick II and I. To capture the nanoprinting images, an experimental optical setup shown in Figure 3A is utilized. As all nanoprinting images are recorded at the sample surface and have the same size as the sample, we utilize an objective with a magnification of 100× to enlarge the images.

The dual-color images of a Chinese character “flower” (sample A) and a picture of sakura (sample B) can be observed under the illumination of a quartz halogen lamp; its color looks orange-red (Figures 3B–3E). When unpolarized white light from light-emitting diode (LED) source is introduced to illuminate the samples, the colors become yellowish (as shown in Figure S2). Due to the spectral difference of the light sources, the dual-color images have different colors. However, all images including the zoom-in views are in clear visual effect under the illumination of a broadband source, which proves the feasibility of encoding a dual-color nanoprinting image.

Next, to decode the information hidden into channel 2 and 3, a red narrow-band filter (the working wavelength is 610 nm with bandwidth of 5 nm), a polarizer, and two analyzers are inserted into the same light path (as shown in Figure 3A). When the transmission axis directions of the polarizer and the analyzer are −45° and 45°, respectively (denoted with white arrows in the upper left corner of Figures 3F and 3H), the reflected nanoprinting images are shown in Figures 3F–3I. The last row presents the experimentally captured nanoprinting images (Figures 3J–3M) by rotating the orthogonal-polarization optical setup clockwise by 22.5°. The experimental results and the zoom-in views indicate that both continuous grayscale images of a “rose” and clear binary-patterns with negligible crosstalk can be observed at the wavelength of 610 nm, which are in good accordance with our design.

In addition, sample A and B are designed to generate the equal continuous gray-images (a “rose”) in channel 2 and different images in channel 1 and 3, which proves that the three channels are controlled independently. Therefore, we can design the three information channels at will, and the information of the three channels is not related and cannot be inferred with each other.

**Figure 4:**
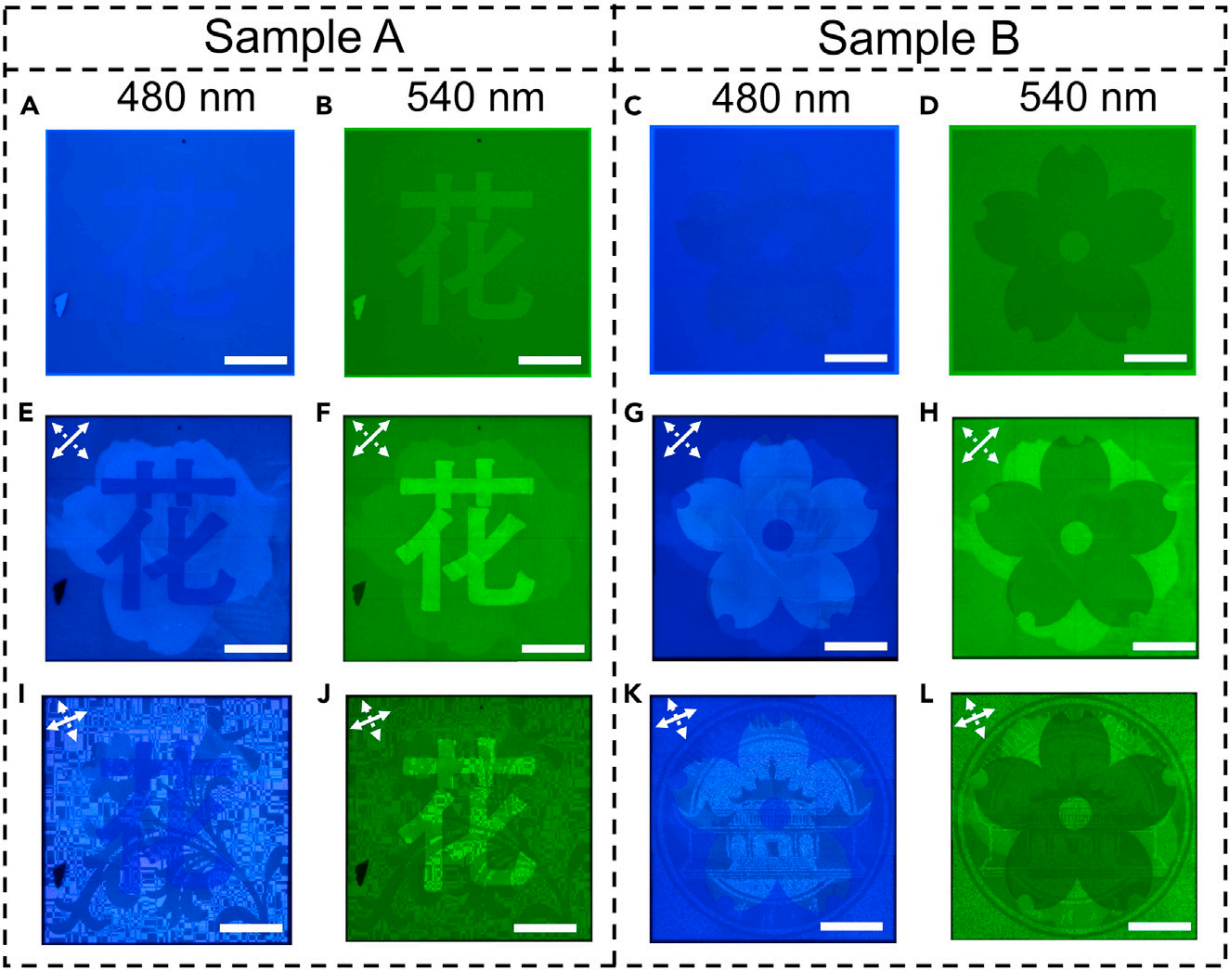
Experimental results of sample A and B under blue and green light illumination (A–D) Nanoprinting images obtained by inserting two narrow-band filters (the working wavelengths are 480 nm and 540 nm, respectively. The bandwidth is 5 nm for both filters) into aforementioned experimental setup of Figure 3. (E–H) Experimentally captured nanoprinting images by tuning the transmission axis directions of the polarizer and the analyzer to −45° and 45°, respectively. (I–L) Experimentally captured nanoprinting images when the orthogonal-polarization optical path is rotated clockwise by an angle of 22.5°. The scale bars are 50 μm. The two different white arrows in the upper left corner of the subfigures show the transmission axis directions of the polarizer and the analyzer, respectively.

At last, to explore the spectral response characteristics of the tri-channel metasurfaces, we capture the nanoprinting images under the illumination of green (*λ* = 540 nm) and blue (*λ* = 480 nm) light, respectively, and the obtained experimental results are shown in Figure 4. Figures 4A–4D show the nanoprinting images captured under the illumination of unpolarized green and blue light. It is obvious that the nanoprinting images obtained in green and blue light illumination appear as the target pattern of channel 1 with different brightness. The main reason is that the reflection of Nanobrick I and II is different at two wavelengths of 480 nm and 540 nm (see STAR Methods for the details of numerical simulations). When an orthogonal-polarization optical path consisting of a polarizer and an analyzer is constructed, the experimentally captured results are shown in the second and third rows of Figure 4. Due to the PCE differences between the Nanobrick I and II at 480 nm and 540 nm, the patterns of channel 2 and 3 are always mixed with the pattern of channel 1, which hinder the information identification.

## Discussion

The proposed “three-in-one” nanoprints provide several technical advantages and have potential applications in many interesting fields. In our design, only two types of nanostructure are employed but we don't bring them together to form a supercell. Instead, each unit-cell consists of either Nanobrick I or II. Because our design is based on single-cell design rather than the widely used supercell design for information multiplexing, our approach has a higher resolution and has potential application in high-density optical storage, as each nanostructure has been multiplexed corresponding to three independent channels.

Secondly, it is interesting to see that the encoded information has to be decoded with quite different optical setups, providing a promising application in designing optical anti-counterfeiting labels. In particular, the information of channel 1, i.e. a dual-color nanoprinting image, is retrieved by a broadband light source without polarization control. And the channel 2 and 3 are decoded by an orthogonal-polarization optical setup with different polarization controls. Therefore, the different illumination conditions can be treated as optical keys to decode the hidden information. In addition, only when the PCE of the two types of nanostructures is equal, can the information hidden in the three channels be completely decoded, which further increases the security of the meta-images. The experimentally measured PCE can reach 11% and 10% for Nanobrick I and II. The efficiency could be improved further by applying more precise fabrication procedures or using low-loss dielectric materials. Because security and counterfeiting difficulties are the fundamental requirements of optical anticounterfeiting labels, our approach with three different keys and three independent images at the nanoscale resolution can significantly improve both the security and counterfeiting difficulty of optical anti-counterfeiting labels.

In summary, we propose a new route of multifunctional light manipulation for separately controlling spectrum and polarization-assisted intensity of incident light, which enlightens the concept of “three-in-one” nanoprints with a single-cell-nanostructured metasurface. Specifically, by combining the spectrum manipulation of varied nanostructures, intensity manipulation governed by Malus law, and the orientation degeneracy of anisotropic nanostructures, a multiplexing metasurface capable of simultaneously and independently recording a dual-color image, a continuous grayscale image, and a binary-pattern is proposed. The experimental results are in good accordance with our design: the metasurface apparently acts as a nanoprint presenting a dual-color image under a broadband light source illumination, while displays two hidden information channels when taking an orthogonal-polarization optical setup and a fixed working wavelength as a decoding key. With advantages such as ultracompactness, high resolution, high security, and difficulty in counterfeiting, the proposed tri-channel metasurfaces have potential applications in optical storage, high-end anti-counterfeiting, information hiding, and many other related fields.

### Limitations of the study

In this work, the contrast of the observed images is not high enough compared with a traditional printing image. Besides, the efficiency should be improved further by applying more precise fabrication procedures or using low-loss dielectric materials.

## STAR★Methods

### Key resources table

**Table:** 

REAGENT or RESOURCE	SOURCE	IDENTIFIER
**Chemicals, peptides, and recombinant proteins**		

PMMA(Polymethyl methacrylate)	Allresist	AR-P 672.06
SOI(Silicon on insulator)	University wafer	3381
Acetone	Sinopharm	67-64-1
Chrome rods	Kurt J.Lesker	EVSCRW2

### Resource availability

#### Lead contact

Any further information and requests for resources and materials should be directed to and will be fulfilled by the Lead Contact, Prof. Guoxing Zheng (gxzheng@whu.edu.cn).

#### Materials availability

This study did not generate new unique reagents.

#### Date and code availability

• Reagents and materials used in the fabrication procedures are listed in the key resources table.
• This paper does not report original code.
• Any additional information required to reanalyze the data reported in this paper is available from the lead contact upon request.

### Method details

#### Theoretical analysis

The Jones matrix of an anisotropic nanostructure with an in-plane orientation *θ* can be expressed as

(Equation 2) $$T\left(\theta \right)=R\left(-\theta \right)T_{0}R\left(\theta \right)=\left[\begin{array}{ll} \text{cos}\theta & -\text{sin}\theta \\ \text{sin}\theta & \text{cos}\theta \end{array}\right].\left[\begin{array}{ll} A & 0\\ 0 & B \end{array}\right].\left[\begin{array}{ll} \text{cos}\theta & \text{sin}\theta \\ -\text{sin}\theta & \text{cos}\theta \end{array}\right],$$

where R(θ) is the rotation matrix, A and B are the complex transmission (or reflection) coefficients of the nanostructure along with the long and short axes, respectively.

If the incident light passes through a polarizer, an anisotropic nanostructure and a bulk-optic analyzer sequentially, the Jones vector of output light can be expressed as

(Equation 3) $$J=\left[\begin{array}{ll} {\text{cos}}^{2}\alpha_{2} & \text{sin}\alpha_{2}\text{cos}\alpha_{2}\\ \text{sin}\alpha_{2}\text{cos}\alpha_{2} & {\text{sin}}^{2}\alpha_{2} \end{array}\right].T\left(\theta \right).\left[\begin{array}{l} \text{cos}\alpha_{1}\\ \text{sin}\alpha_{1} \end{array}\right],$$

where $\alpha_{1}$ and $\alpha_{2}$ represent the directions of transmission axis of the polarizer and analyzer, respectively. If the light intensity after the polarizer is $I_{0}$, we can deduce the expression of output light intensity according to Equation 3 as

(Equation 4) $$I=I_{0}{\left[\frac{A-B}{2}\cos\left(2\theta -\alpha_{2}-\alpha_{1}\right)+\frac{A+B}{2}\cos\left(\alpha_{2}-\alpha_{1}\right)\right]}^{2}.$$

We find that any anisotropic nanostructure (A≠B) can be used for a continuous intensity modulation when the light intensity $I_{0}$, the transmission axes of the polarizer and analyzer are unambiguously given.

When the transmission axis of the polarizer is perpendicular to the transmission axis of the analyzer, we can simplify Equation 4 as

(Equation 5) $$I_{1}=I_{0}{\left(\frac{A-B}{2}\right)}^{2}{\cos\;}^{2}\left(2\theta -\alpha_{2}-\alpha_{1}\right).$$

Next, if we rotate the nanostructure by an angle such as 22.5° around its optical axis, the corresponding output light intensity is changed to

(Equation 6) $$I_{2}=I_{0}{\left(\frac{A-B}{2}\right)}^{2}{\cos\;}^{2}\left(2\theta -\alpha_{2}-\alpha_{1}-45{}^{\circ}\right).$$

Specifically, if the nanobrick acts as an ideal half-wave plate (i.e., *A*=1 and *B*=-1) and $\alpha_{2}=-\alpha_{1}=45{}^{\circ}$, we can simplify Equations 5 and 6 as

(Equation 7) $$I_{1}=I_{0}\;{\cos\;}^{2}\left(2\theta \right)$$

and

(Equation 8) $$I_{2}=I_{0}\;{\cos\;}^{2}\left(2\theta -45{}^{\circ}\right).$$

From Equations 7 and 8 we can find that there are four orientation candidates in its defined interval of [0°, 180°] to generate an equal output intensity, which can be named as the orientation degeneracy of anisotropic nanostructures. The orientation degeneracy provides a new degree of freedom, which will benefit for encoding a continuous grayscale image into channel 2 and an independent two-step image into channel 3 with a single piece of metasurface. More details about the working principle have been demonstrated in the main text.

#### Numerical simulations

A unit-cell of the metasuface is shown in Figure S3. We employed CST STUDIO SUITE software to design and simulate two types of nanobrick unit-cells. LP light with a polarization angle of 45° was normally incident onto a nanobrick and the periodic boundary conditions were utilized. Hence, the spectra of reflectance and PCE were retrieved from the simulations, as shown in Figures S3B and S3C. In our design, a SOI material with top silicon of 220 nm thick was used to construct the single-cell metasurface. The performance of the two types of silicon nanobricks was optimized by sweeping the width and length, while fixing the height at 220 nm, which was determined by the thickness of the top layer of the SOI wafer we chose. In order to reduce the near-field coupling effect between adjacent nanobricks and considering the fabrication difficulty comprehensively, the unit size C was carefully chosen to be 400 nm.

#### Sample fabrication

The samples were fabricated with SOI material (top silicon of 220 nm and silicon dioxide of 2 μm) by employing the standard electron beam lithography process (EBL). First, using a thermal evaporator, we deposited a 30 nm Cr thin film onto the SOI material. Subsequently, it was dipped in acetone and washed with ultrasonic waves. Following this, the reactive ion etching (RIE) was used to remove the Cr-free part. Final, the desired nanostructures were obtained by using a Cr etchant to eliminate the remained Cr mask. More details about the fabrication process for the SOI nanobrick based metasurfaces are shown in Figure S4.

## References

[bib1] Cheng F., Gao J., Luk T.S., Yang X. (2015). Structural color printing based on plasmonic metasurfaces of perfect light absorption. Sci. Rep..

[bib2] Chen W., Zhu A., Sanjeev V., Khorasaninejad M., Shi Z., Lee E., Capasso F. (2018). A broadband achromatic metalens for focusing and imaging in the visible. Nat. Nanotechnol..

[bib3] Chen Y., Yang X., Gao J. (2018). Spin-controlled wavefront shaping with plasmonic chiral geometric metasurfaces. Light Sci. Appl..

[bib4] Chen Y., Gao J., Yang X. (2019). Chiral grayscale imaging with plasmonic metasurfaces of stepped nanoapertures. Adv. Opt. Mater..

[bib5] Chen Y., Yang X., Gao J. (2019). 3D Janus plasmonic helical nanoapertures for polarization-encrypted data storage. Light Sci. Appl..

[bib6] Dai Q., Li Z., Deng L., Zhou N., Deng J., Tao J., Zheng G. (2020). Single-size nanostructured metasurface for dual-channel vortex beam generation. Opt. Lett..

[bib7] Dai Q., Guan Z., Chang S., Deng L., Tao J., Li Z.-Y., Li Z., Yu S., Zheng G., Zhang S. (2020). A single-celled tri-functional metasurface enabled with triple manipulations of light. Adv. Funct. Mater..

[bib8] Dai Q., Zhou N., Deng L., Deng J., Li Z., Zheng G. (2020). Dual-channel binary gray-image display enabled with malus-assisted metasurfaces. Phys. Rev. Appl..

[bib9] Deng J., Yang Y., Tao J., Deng L., Liu D., Guan Z., Li G., Li Z., Yu S., Zheng G. (2019). Spatial frequency multiplexed meta-holography and meta-nanoprinting. ACS Nano.

[bib10] Deng Z., Jin M., Ye X., Wang S., Shi T., Deng J., Mao N., Cao Y., Guan B., Alu A. (2020). Full-color complex-amplitude vectorial holograms based on multi-freedom metasurfaces. Adv. Funct. Mater..

[bib11] Deng L., Deng J., Guan Z., Tao J., Chen Y., Yang Y., Zhang D., Tang J., Li Z., Li Z. (2020). Malus-metasurface-assisted polarization multiplexing. Light Sci. Appl..

[bib12] Deng J., Deng L., Guan Z., Tao J., Li G., Li Z., Li Z., Yu S., Zheng G. (2020). Multiplexed anticounterfeiting meta-image displays with single-sized nanostructures. Nano Lett..

[bib13] Dong Z., Ho J., Yu Y.F., Fu Y.H., Paniagua-Dominguez R., Wang S., Kuznetsov A., Yang J.K. (2017). Printing beyond sRGB color gamut by mimicking silicon nanostructures in free-space. Nano Lett..

[bib14] Ellenbogen T., Seo K., Crozier K.B. (2012). Chromatic plasmonic polarizers for active visible color filtering and polarimetry. Nano Lett..

[bib15] Fan Q., Liu M., Zhang C., Zhu W., Wang Y., Lin P., Yan F., Chen L., Lezec H.J., Lu Y. (2020). Independent amplitude control of arbitrary orthogonal states of polarization via dielectric metasurfaces. Phys. Rev. Lett..

[bib16] Fang B., Li H., Zhu S., Li T. (2020). Second-harmonic generation and manipulation in lithium niobate slab waveguides by grating metasurfaces. Photonics Res..

[bib17] Fu R., Li Z., Zheng G., Chen M., Yang Y., Tao J., Wu L., Deng Q. (2019). Reconfigurable step-zoom metalens without optical and mechanical compensations. Opt. Express.

[bib18] Fu R., Deng L., Guan Z., Chang S., Tao J., Li Z., Zheng G. (2020). Zero-order-free meta-holograms in a broadband visible range. Photonics Res..

[bib19] Goh X.M., Zheng Y., Tan S.J., Zhang L., Kumar K., Qiu C.W., Yang J.K. (2014). Three-dimensional plasmonic stereoscopic prints in full colour. Nat. Commun..

[bib20] Guo Y., Pu M., Zhao Z., Wang Y., Jin J., Gao P., Li X., Ma X., Luo X. (2016). Merging geometric phase and plasmon retardation phase in continuously shaped metasurfaces for arbitrary orbital angular momentum generation. ACS Photonics.

[bib21] Guo J., Wang T., Quan B., Zhao H., Gu C., Li J., Wang X., Situ G., Zhang Y. (2019). Polarization multiplexing for double images display. Opto-Electron. Adv..

[bib22] Hu Y., Luo X., Chen Y., Liu Q., Li X., Wang Y., Na L., Duan H. (2019). 3D-Integrated metasurfaces for full-colour holography. Light Sci. Appl..

[bib23] Hu Y., Liu X., Jin M., Tang Y., Zhang X., Li K.F., Zhao Y., Li G., Zhou J. (2021). Dielectric metasurface zone plate for the generation of focusing vortex beams. PhotoniX.

[bib24] Jang J., Jeong H., Hu G., Qiu C.W., Nam K.T., Rho J. (2019). Kerker-conditioned dynamic cryptographic nanoprints. Adv. Opt. Mater..

[bib25] Jiang Q., Jin G., Cao L. (2019). When metasurface meets hologram: principle and advances. Adv. Opt. Photonics.

[bib26] Jung C., Yang Y., Jang J., Badloe T., Lee T., Mun J., Moon S.-W., Rho J. (2020). Near-zero reflection of all-dielectric structural coloration enabling polarization-sensitive optical encryption with enhanced switchability. Nanophotonics.

[bib27] Kim I., Jeong H., Kim J., Yang Y., Lee D., Badloe T., Kim G., Rho J. (2021). Dual-band operating metaholograms with heterogeneous meta-atoms in the visible and near-infrared. Adv. Opt. Mater..

[bib28] Kim I., Jang J., Kim G., Lee J., Badloe T., Mun J., Rho J. (2021). Pixelated bifunctional metasurface-driven dynamic vectorial holographic color prints for photonic security platform. Nat. Commun..

[bib29] Koirala S., Lee S., Choi D.Y. (2018). Highly transmissive subtractive color filters based on an all-dielectric metasurface incorporating TiO_2_ nanopillars. Opt. Express.

[bib30] Li Z., Zheng G., Li S., Deng Q., Zhao J., Ai Y. (2015). All-silicon nanorod-based Dammann gratings. Opt. Lett..

[bib31] Li Z., Butun S., Aydin K. (2015). Large-area, lithography-free super absorbers and color filters at visible frequencies using ultrathin metallic films. ACS Photonics.

[bib32] Li Z., Kim I., Zhang L., Mehmood M.Q., Anwar M.S., Saleem M., Dasol L., Nam K., Zhang S., Luk’yanchuk B. (2017). Dielectric meta-holograms enabled with dual magnetic resonances in visible light. ACS Nano.

[bib33] Li Z., Dai Q., Mehmood M.Q., Hu G., Luk’yanchuk B., Tao J., Hao C., Kim I., Jeong H., Zheng G. (2018). Full-space cloud of random points with a scrambling metasurface. Light Sci. Appl..

[bib34] Li Z., Yu S., Zheng G. (2020). Advances in exploiting the degrees of freedom in nanostructured metasurface design: from 1 to 3 to more. Nanophotonics.

[bib35] Li Z., Chen C., Guan Z., Tao J., Chang S., Dai Q., Xiao Y., Cui Y., Wang Y., Yu S. (2020). Three-channel metasurfaces for simultaneous meta-holography and meta-nanoprinting: a single-cell design approach. Laser Photonics Rev..

[bib36] Li L., Liu Z., Ren X., Wang S., Su V.-C., Chen M.-K., Chu C.H., Kuo H.Y., Liu B., Zang W. (2020). Metalens-array–based high-dimensional and multiphoton quantum source. Science.

[bib37] Li J., Wang Y., Chen C., Fu R., Zhou Z., Li Z., Zheng G., Yu S., Qiu C.-W., Zhang S. (2021). From lingering to rift: metasurface decoupling for near- and far-field functionalization. Adv. Mater..

[bib38] Li Z., Dai Q., Deng L., Li G., Zheng G. (2021). Structural-color nanoprinting with hidden watermarks. Opt. Lett..

[bib39] Li Z., Ren R., Deng J., Deng L., Li G., Zheng G. (2021). Non-orthogonal-polarization multiplexed metasurfaces for tri-channel gray-imaging. Opt. Express.

[bib40] Liu L., Wang H., Han Y., Lu X., Lv H., Teng S. (2019). Color filtering and displaying based on hole array. Opt. Commun..

[bib41] Liu M., Zhu W., Huo P., Feng L., Song M., Zhang C., Chen L., Lezec H.J., Lu Y., Agrawal A. (2021). Multifunctional metasurfaces enabled by simultaneous and independent control of phase and amplitude for orthogonal polarization states. Light Sci. Appl..

[bib42] Liang C., Deng L., Dai Q., Li Z., Zheng G., Guan Z., Li G. (2021). Single-celled multifunctional metasurfaces merging structural-color nanoprinting and holography. Opt. Express.

[bib43] Olson J., Manjavacas A., Basu T., Huang D., Schlather A.E., Zheng B., Halas N., Nordlander P., Link S. (2015). High chromaticity aluminum plasmonic pixels for active liquid crystal displays. ACS Nano.

[bib44] Proust J., Bedu F., Gallas B., Ozerov I., Bonod N. (2016). All-dielectric colored metasurfaces with silicon Mie resonators. ACS Nano.

[bib45] Ren H., Fang X., Jang J., Bürger J., Rho J., Maier S.A. (2020). Complex-amplitude metasurface-based orbital angular momentum holography in momentum space. Nat. Nanotechnol..

[bib46] Shan X., Deng L., Dai Q., Zhou Z., Liang C., Li Z., Zheng G. (2020). Silicon-on-insulator based multifunctional metasurface with simultaneous polarization and geometric phase controls. Opt. Express.

[bib47] Solntsev A.S., Agarwal G.S., Kivshar Y.S. (2021). Metasurfaces for quantum photonics. Nat. Photonics.

[bib48] Sun S., Zhou Z., Zhang C., Gao Y., Duan Z., Xiao S., Song Q. (2017). All-dielectric full-color printing with TiO_2_ metasurfaces. ACS Nano.

[bib49] Tan S.J., Zhang L., Zhu D., Goh X.M., Wang Y.M., Kumar K., Qiu C.W., Yang J.K. (2014). Plasmonic color palettes for photorealistic printing with aluminum nanostructures. Nano Lett..

[bib50] Tseng M.L., Yang J., Semmlinger M., Zhang C., Nordlander P., Halas N.J. (2017). Two-dimensional active tuning of an aluminum plasmonic array for full-spectrum response. Nano Lett..

[bib51] Wang S., Wu P., Su V., Lai Y., Chen M., Kuo H., Chen B., Chen Y., Huang T., Wang J. (2018). A broadband achromatic metalens in the visible. Nat. Nanotechnol..

[bib52] Wang Y., Fan Q., Xu T. (2021). Design of high efficiency achromatic metalens with large operation bandwidth using bilayer architecture. Opto-Electron. Adv..

[bib53] Xu T., Wu Y.K., Luo X., Guo L.J. (2010). Plasmonic nanoresonators for high-resolution colour filtering and spectral imaging. Nat. Commun..

[bib54] Yang B., Liu W., Li Z., Cheng H., Chen S., Tian J. (2018). Polarization-sensitive structural colors with hue-and-saturation tuning based on all-dielectric nanopixels. Adv. Opt. Mater..

[bib55] Yang W., Xiao S., Song Q., Liu Y., Wu Y., Wang S., Yu J., Han J., Tsai D.-P. (2020). All-dielectric metasurface for high-performance structural color. Nat. Commun..

[bib56] Yang Y., Yoon G., Park S., Namgung S.D., Badloe T., Nam K.T., Rho J. (2021). Revealing structural disorder in hydrogenated amorphous silicon for a low-loss photonic platform at visible frequencies. Adv. Mater..

[bib57] Yoon G., Lee D., Nam K.T., Rho J. (2018). “Crypto-Display” in dual-mode metasurfaces by simultaneous control of phase and spectral responses. ACS Nano.

[bib58] Yue F., Zhang C., Zang X.F., Wen D., Gerardot B.D., Zhang S., Chen X. (2018). High-resolution grayscale image hidden in a laser beam. Light Sci. Appl..

[bib59] Zhang Y., Shi L., Hu D., Chen S., Xie S., Lu Y., Cao Y., Zhu Z., Jin L., Guan B.O. (2019). Full-visible multifunctional aluminium metasurfaces by in situ anisotropic thermoplasmonic laser printing. Nanoscale Horiz..

[bib60] Zhang F., Pu M., Gao P., Jin J., Li X., Guo Y., Ma X., Luo J., Yu H., Luo X. (2020). Simultaneous full-color printing and holography enabled by centimeter-scale plasmonic metasurfaces. Adv. Sci..

[bib61] Zheng G., Wu W., Li Z., Zhang S., Mehmood M.Q., Li S. (2017). Dual field-of-view step-zoom metalens. Opt. Lett..

[bib62] Zheng P., Dai Q., Li Z., Ye Z., Xiong J., Liu H.-C., Zheng G., Zhang S. (2021). Metasurface-based key for computational imaging encryption. Sci. Adv..

[bib63] Zhu L., Liu X., Sain B., Wang M., Schlickriede C., Tang Y., Deng J., Li K., Yang J., Holynski M. (2020). A dielectric metasurface optical chip for the generation of cold atoms. Sci. Adv..

