# Supplementary material for: Metasurface-enabled three-in-one nanoprints by multifunctional manipulations of light
Source: iScience. 2021 Nov 26;24(12):103510. doi: 10.1016/j.isci.2021.103510 (PMC8669004; doi:10.1016/j.isci.2021.103510)
Supplement: Document S1. Figures S1–S4 [file mmc1.pdf]

iScience, Volume *24*

Supplemental information

Metasurface-enabled three-in-one nanoprints by multifunctional manipulations of light

Zile Li, Liangui Deng, Juan Deng, Zhixue He, Jin Tao, Guoxing Zheng, and Shaohua Yu

Supplemental Information

Figure S1: Optical path for decoding tri-channel information

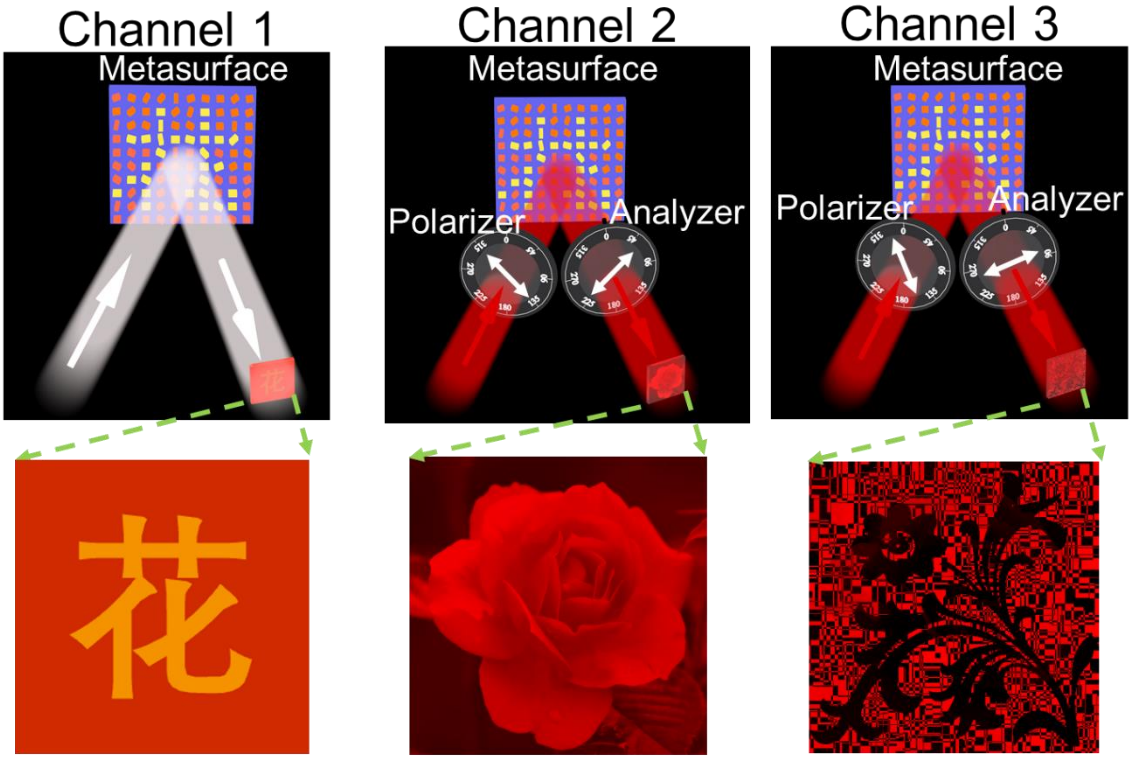

Figure S1. Schematic illustration of decoding tri-channel information. Channel 1: The reflective dual-color image can be generated under white light illumination. Channel 2: A continuous grayscale image can be decoded at the working wavelength of 610 nm with the polarizer's and analyzer's transmission axes of -45° and 45°, respectively. Channel 3: An additional binary-pattern appears at the working wavelength of 610 nm with the polarizer's and analyzer's transmission axes of -22.5° and 67.5°, respectively. Related to Figure 1.

Figure S2: Dual-color images under white LED illumination

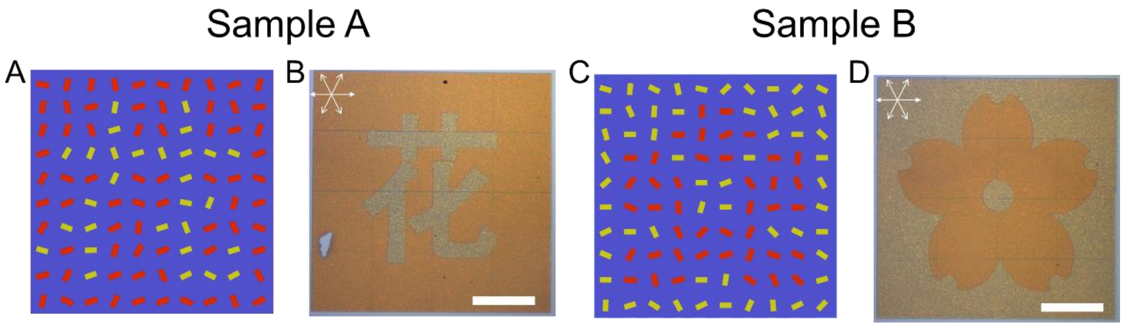

Figure S2. Design and experimental results of sample A and B under the illumination of a light-emitting diode (LED) source. (A, C) The background and target parts of sample A are designed with Nanobrick II and I respectively, whereas the situation of sample B is the opposite of sample A. (B, D) Experimentally captured nanoprinting-images. The background and target pattern color of sample A are opposite to those of sample B. Due to the spectral difference of the light sources, its color looks yellowish compared to a quartz halogen lamp. The scale bar is 50 μm. Related to Figure 3.

Figure S3: Numerical Simulations of a unit-cell

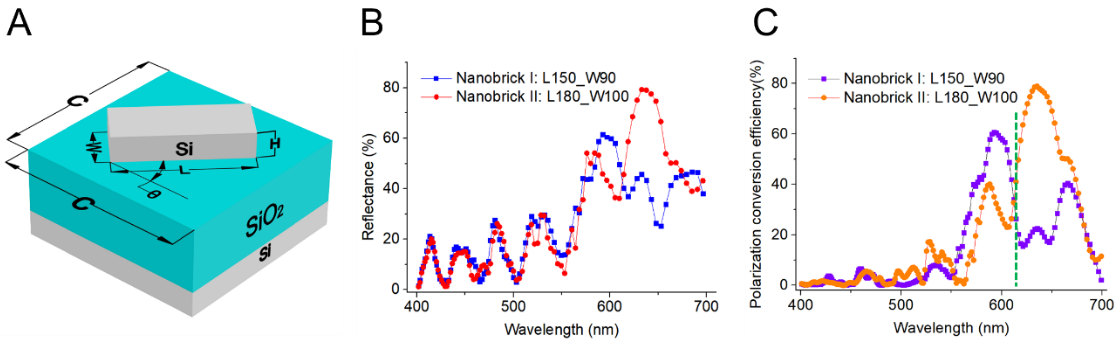

Figure S3. Illustration of a unit-cell structure, spectral response and the PCE of the tri-channel metasurfaces. (A) Schematic of a silicon-on-insulator (SOI) nanobrick, in which the thicknesses of the crystalline silicon and the silicon dioxide (SiO_2) layer are 220 nm and 2 μm respectively. (B) Simulated reflective spectra of the two types of nanobricks, named as Nanobrick **I** and **II**. The spectral differences will benefit for generating a dual-color meta-image. (C) PCE of Nanobrick **I** and **II** for LP light illumination. At the working wavelength of 610 nm, the PCE of the two nanobricks is equal, which contributes to design hidden dual-channel information. Related to Numerical Simulations in the Methods.

Figure S4: Fabrication process for SOI nanobricks

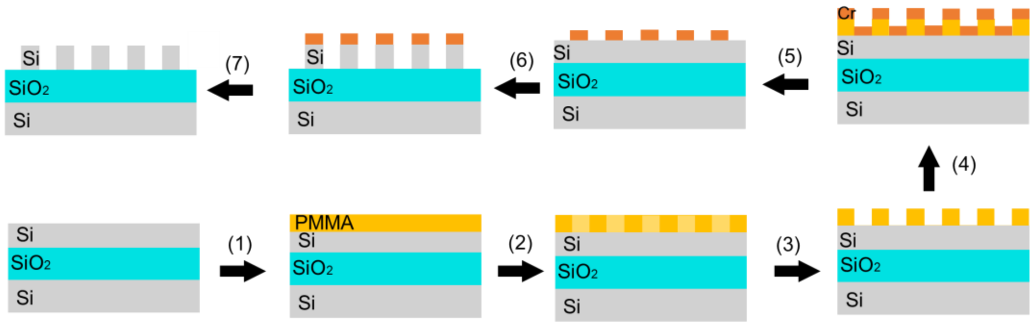

Figure S4. Fabrication process for the SOI nanobricks. (1) a layer of PMMA (polymethyl methacrylate) electron beam resist was coated on the SOI substrate after cleaning, dehydration and baking; (2) the nanobrick pattern area was exposed using the electron beam; (3) the exposed portion of the resist was dissolved by using developer; (4) a 30 nm Cr film was deposited on the sample by using Electron-beam Evaporator; (5) the sample was immersed in acetone and the excess resist and Cr film were removed with ultrasonic waves; (6) the Cr-free portion of silicon was removed by using reactive ion etching (RIE); (7) the remaining Cr mask was eliminated by Cr etchant. Related to Sample Fabrication in the Methods.
